# Reduction of osteoarthritis severity in the temporomandibular joint of rabbits treated with chondroitin sulfate and glucosamine

**DOI:** 10.1371/journal.pone.0231734

**Published:** 2020-04-15

**Authors:** Felipe Ernesto Artuzi, Edela Puricelli, Carlos Eduardo Baraldi, Alexandre Silva Quevedo, Deise Ponzoni

**Affiliations:** 1 School of Dentistry/Federal University of Rio Grande do Sul (UFRGS), Porto Alegre, Rio Grande do Sul, Brazil; 2 Oral and Maxillofacial Surgery Unit/ Clinical Hospital of Porto Alegre (HCPA), School of Dentistry/Federal University of Rio Grande do Sul (UFRGS), Porto Alegre, Rio Grande do Sul, Brazil; UMR 7365 CNRS UL, FRANCE

## Abstract

Osteoarthritis is a degenerative disease that causes substantial changes in joint tissues, such as cartilage degeneration and subchondral bone sclerosis. Chondroitin sulfate and glucosamine are commonly used products for the symptomatic treatment of osteoarthritis. The aim of the present study was to investigate the effects of these products when used as structure-modifying drugs on the progression of osteoarthritis in the rabbit temporomandibular joint. Thirty-six New Zealand rabbits were divided into 3 groups (n = 12/group): control (no disease); osteoarthritis (disease induction); and treatment (disease induction and administration of chondroitin sulfate and glucosamine). Osteoarthritis was induced by intra-articular injection of monosodium iodoacetate. Animals were killed at 30 and 90 days after initiation of therapy. The treatment was effective in reducing disease severity, with late effects and changes in the concentration of glycosaminoglycans in the articular disc. The results indicate that chondroitin sulfate and glucosamine may have a structure-modifying effect on the tissues of rabbit temporomandibular joints altered by osteoarthritis.

## Introduction

Osteoarthritis (OA) is a severe joint disease that can affect the temporomandibular joint (TMJ), causing pain and functional limitations that compromise quality of life. The main pathologic features of temporomandibular joint osteoarthritis (TMJ-OA) are cartilage degeneration and subchondral bone sclerosis [1]. Optimal treatment involves altering the natural history of OA and reducing symptoms, inflammatory levels, and degenerative effects on cartilages and joint tissues [2].

Chondroitin sulfate (CS) and glucosamine (G) are commonly used as medicines or nutraceuticals to control the symptoms of OA, especially pain, stiffness, and decreased functional capacity of the affected joint [3–7]. Although many studies have shown significant treatment effects, the actual efficacy of CS and G treatment compared with other treatments is still controversial [8]. In humans, the positive effects of CS and G when used as disease-modifying OA drugs (DMOADs), whether alone or in combination (CS+G), have been observed during long-term clinical studies and evaluated by imaging studies [4,9,10]. Experimental animal studies testing CS and G, however, have yielded conflicting results and often assess the effects in knee OA [11–16].

The TMJ fibrocartilage differs structurally and functionally from hyaline cartilage [17], suggesting that the mechanisms of action and modulatory effects of CS+G on OA might not be the same [18]. The main distinguishing feature is that the mandibular condyle is covered by a thin layer of fibrous connective tissue containing mesenchymal cells that differentiate into chondrocytes, thus being regarded as fibrocartilage [19]. Fibrocartilage, a secondary tissue derived from perivascular osteogenic cells, has a denser extracellular matrix than hyaline cartilage consisting of fibrous connective tissue that is primarily composed of glycosaminoglycans (GAGs) and type I collagen fibers. The predominance of this type of collagen is characteristic of fibrocartilage and associated with the need to support mechanical loading [20]. Collagen fibers are directly related to the tensile strength property of cartilages, while proteoglycans with their GAG side chains allow for tissue expansion due to osmotic pressure [21]. Anterior disc displacement and OA trigger the release of cytokines and growth factors in TMJ synovial fluid, including tumor necrosis factor alpha (TNF-α), interleukin 1 beta (IL-1β), interleukin 6 (IL-6), interleukin 8 (IL-8), and prostaglandin E_2_ (PGE_2_). Cytokines participate in several inflammatory processes and induce protease synthesis and release, which may cause proteoglycan and collagen depletion, thus leading to the cartilage degradation observed in OA [22].

Although fibrocartilage has limited regenerative capacity, important advances have been made in the processes of growth factor modulation and cell differentiation involving chondrogenesis in the repair of cartilage and subchondral bone tissue in TMJ-OA [23,24]. Some studies suggest that CS+G or CS combined with hyaluronic acid may stimulate the differentiation of progenitor cells, contributing to a more rapid and effective tissue repair of joint defects [18,25]. It is well established that insulin-like growth factors (IGF), transforming growth factors (TGF), fibroblast growth factors (FGF), bone morphogenetic proteins (BMP), parathyroid hormone-related peptide (PTHrP), members of the hedgehog *(Ihh)* family, and the *Wnt* pathway provide important signals for the regulation of chondrocyte proliferation, differentiation, and maturation during chondrogenesis. In response to external stimuli, articular cartilage would be able to respond to adaptive changes by modulating these factors, resulting in multidirectional possibilities of condylar growth and remodeling [17,26].

In response to mechanical stress, the TMJ disc has the ability to modify the synthesis of GAGs, especially of chondroitin 6-sulfate (CS6), hyaluronic acid, and dermatan sulfate. As a result, the biochemical properties of the disc are also continually modified [27]. Tissue response, however, is dependent on the magnitude and duration of compressive forces and the individual’s adaptive capacity [28]. Total GAG concentration in the TMJ disc ranges from 0.6 to 10% of the dry weight [29]. Collagen accounts for approximately 30% of total disc wet weight, most of which is type I collagen [30]. In an interspecies comparison, the GAG concentration of rabbit TMJ discs was higher than that of human discs, but collagen content was similar—although the human disc was significantly stiffer and stronger that the rabbit disc [31].

Clinical trials evaluating the use of CS+G for the symptomatic treatment of TMJ dysfunction have reported different levels of efficacy [32–35]. However, to date, no study has assessed the effects of CS and G used as DMOADs in TMJ-OA. Therefore, the present study investigated the 30- and 90-day effects of CS+G on the progression of TMJ-OA induced in rabbits by intra-articular injection of monosodium iodoacetate (MIA). The histologic appearance of TMJ cartilage was evaluated and GAGs were quantified in the TMJ cartilage and discs after 30 and 90 days of treatment. The hypothesis was that the induction of TMJ-OA would reduce the total amount of GAGs in the articular disc and cartilage via breakdown of the extracellular matrix and that CS+G would restore the lost GAGs and have anabolic effects on cartilage, reversing the degeneration caused by OA.

## Materials and methods

### Experimental design

Thirty-six 4-month-old male New Zealand rabbits (*Oryctolagus cuniculus* L.) weighing 3 to 4 kg each were used in the study. The animals were housed in individual cages under a 12 h/12 h light/dark cycle, temperature of 21±1°C, and relative humidity of 40–60% and given water, rabbit chow, and green leaves *ad libitum*. The study was performed in accordance with Brazilian law no. 11794/2008, which establishes the guidelines for the care and use of laboratory animals, and was approved by the Ethics Committee for the Use of Animals in Research of Hospital de Clínicas de Porto Alegre (HCPA), approval no. 160238. All rabbits used in the study came from a single breeding farm, which is a registered supplier to HCPA. The rabbits’ health conditions were monitored by a veterinarian during the entire experiment. Rabbits were weighed weekly to calculate the CS+G dose and to control for potential diseases. For OA induction, the animals were anesthetized and received analgesia. Because treatment involved a simple and noninvasive procedure, the animals were restrained for treatment administration. For euthanasia, the animals were anesthetized to avoid pain and minimize suffering.

**Fig 1:**
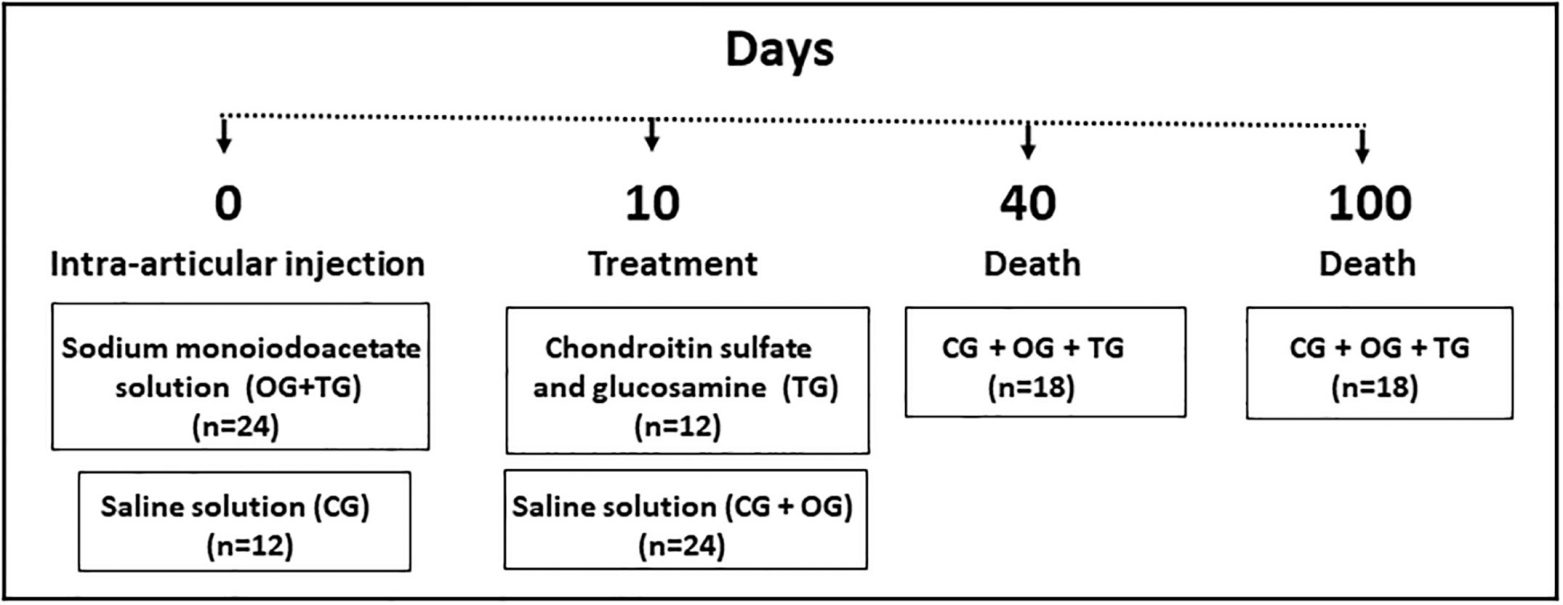
Study design.

Rabbits were randomly divided into 3 groups of 12 animals each: control (CG, no OA induction); osteoarthritis (OG, OA induction); and treatment (TG, OA induction and CS+G treatment). The animals were anesthetized with ketamine (10 mg/kg) and midazolam (1 mg/kg) injected intramuscularly. TMJ-OA was induced in OG and TG rabbits by intra-articular injection of 50 μL of 10 mg/mL MIA into the TMJ bilaterally. CG animals were injected with 50 μL of saline (Fig 1). A 30G needle (8 mm x 0.3 mm) was used to inject the solutions, and the injection site was determined at 5 mm above and distal to the posterior border of the zygomatic process, according to a previously established model [36].

### Treatment

Treatment was started 10 days after induction of OA. TG animals were given a subcutaneous injection of 0.1 mL/kg of Condroton^®^ every 3 days, which corresponds to approximately 7.5 mg/kg of CS and 7.5 mg/kg of G. Doses were calculated according to the recommended dose [37]. Rabbits were weighed weekly and drug doses were adjusted accordingly. CG and OG animals were injected with saline at the same dose of 0.1 mL/kg. Subsequently, according to predetermined time points (40 and 100 days after induction of OA), animals were anesthetized with a combination of ketamine (20 mg/kg), meperidine (3 mg/kg), and midazolam (1 mg/kg) injected intramuscularly and killed with an overdose of propofol (5 mg/kg) followed by intravenous injection of potassium chloride (1 mL/kg).

### Histologic analysis

The right TMJs were removed, fixed in 10% buffered formalin, decalcified in 5% nitric acid, and sectioned in the sagittal plane. The sections were stained with safranin O/fast-green and hematoxylin and eosin (H&E) for histologic analysis. To assess the degree of joint degeneration, the slides were analyzed qualitatively by an experienced pathology professor, who was blinded to group allocation, using the OA grading system proposed by Pritzker et al. [38]. In this system, the OA score represents an assessment of OA severity, ranging from 0 (absence of OA) to 6 (maximum OA severity).

### Sample preparation and GAG quantification

The left TMJs were dissected to separate the discs and fibrocartilage. Specimens were obtained from the anterior and central regions, including the lateral aspects, of the articular fibrocartilage and discs. The specimens were weighed and sectioned in half in the coronal plane; 10 mg of articular disc and 5 mg of fibrocartilage were obtained from each specimen and stored at −80°C. GAGs were quantified using the method proposed by De Jong et al. [39], with modifications. The specimens were fragmented with a scalpel, ground, and incubated in a dry bath at 60°C for 24 h in a solution consisting of 150 μL of 50 mmol/mL phosphate buffer, pH 6.5, 0.24 g/L L-cysteine, 0.4% 0.5 M EDTA, and 0.0607 mg/mL papain (15 μL of papain for discs and 9 μL for cartilage). GAGs were separated by adding 300 μL of chloroform, followed by centrifugation at 10,000 g (9,000 rpm) for 15 min at 4°C. The supernatant was then collected for analysis. GAG content was quantified by dimethylmethylene blue (DMB) assay, in which 5 μL of the sample was mixed with a DMB solution (0.3 mol/L DMB with hydroxymethyl aminomethane and 2 mol/l Tris), and absorbance was read at 530 nm (Spectramax M3 multi-mode microplate reader, Molecular Devices, China). The results were expressed as μg GAGs/mg wet weight.

### Statistical analysis

Median scores obtained in histologic analysis were compared by the nonparametric Kruskal-Wallis test followed by Dunn’s post hoc test. Mean GAG concentrations were compared between all conditions (groups and time points) by analysis of variance (ANOVA) followed by Fisher’s least significant difference (LSD) test. Student’s *t* test for independent samples was used to compare GAG concentrations between groups (without considering the time points). SPSS, version 21.0 (SPSS Inc., Chicago, IL, USA), was used for data analysis. The level of significance was set at 5% (*p<0*.*05*).

## Results

**Fig 2:**
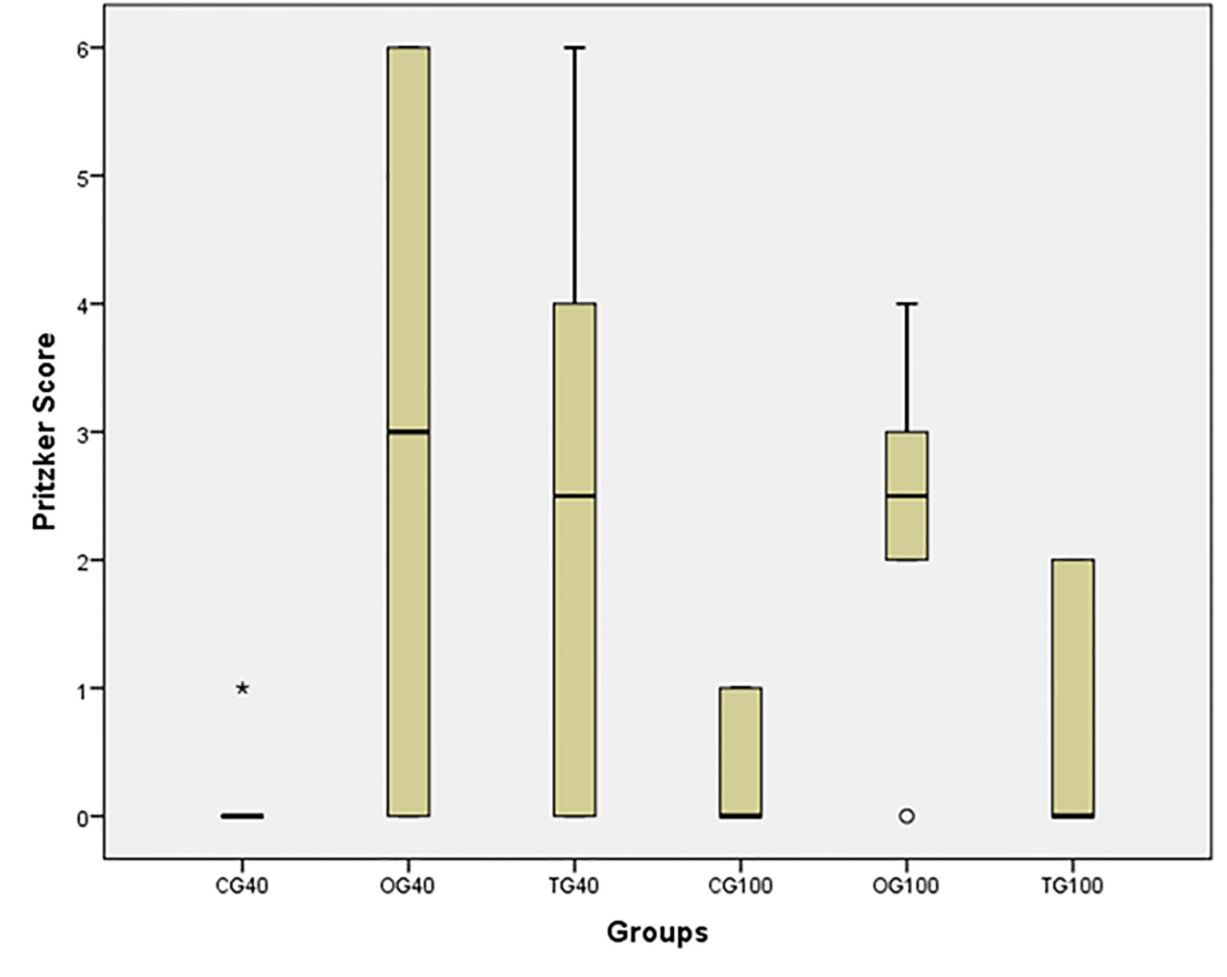
Box plot of median and interquartile range of osteoarthritis severity scores for each group at each assessment time point. Osteoarthritis severity was assessed using the grading system proposed by Pritzker et al. [38], where 0 indicates ‘absence’ and 6 ‘maximum severity’. Control group showed no signs of osteoarthritis at 40 days and insignificant or incipient disease at 100 days. Osteoarthritis group had intermediate median scores at both time points, indicating that disease severity remained stable throughout the study. At 40 days, median scores were similar for the treatment and osteoarthritis groups, but at 100 days the treatment group had lower scores, indicating that treatment with chondroitin sulfate and glucosamine was effective (CG_40_: control group at 40 days, OG_40_: osteoarthritis group at 40 days, TG_40_: treatment group at 40 days, CG_100_: control group at 100 days, OG_100_: osteoarthritis group at 100 days, TG_100_: treatment group at 100 days).

The results of OA severity grading are shown in Fig 2. There was a significant difference in OA severity between the 3 groups (*x*^*2*^ = *12*.*239*, *p = 0*.*032*). CG and OG differed significantly at both 40-day and 100-day assessment time points (CG_40_<OG_40_, *p = 0*.*049*; and CG_100_<OG_100_, *p = 0*.*019*), indicating stable disease maintenance. There was no significant difference between CG and TG at any time point (CG_40_ = TG_40_, *p = 0*.*050*; and CG_100_ = TG_100_, *p = 0*.*702*). This result may indicate the late effectiveness of CS+G treatment. There was no significant difference between TG and OG at 40 days (*p = 0*.*741*), indicating that treatment was not effective after 30 days of administration. At 100 days, TG showed a significantly lower median score than OG (*p = 0*.*042*), indicating that treatment was effective in reversing the degeneration caused by OA. However, treatment effect did not change over time (TG_40_ = TG_100_, *p = 0*.*121*). In OG, OA severity remained unchanged from day 40 to day 100 *(p = 0*.*621)*. Animals in the CG showed no significant degenerative changes in joint tissues over time (CG_40_ = CG_100_, *p = 0*.*523*). (S1 Table).

### Histologic evaluation

**Fig 3:**
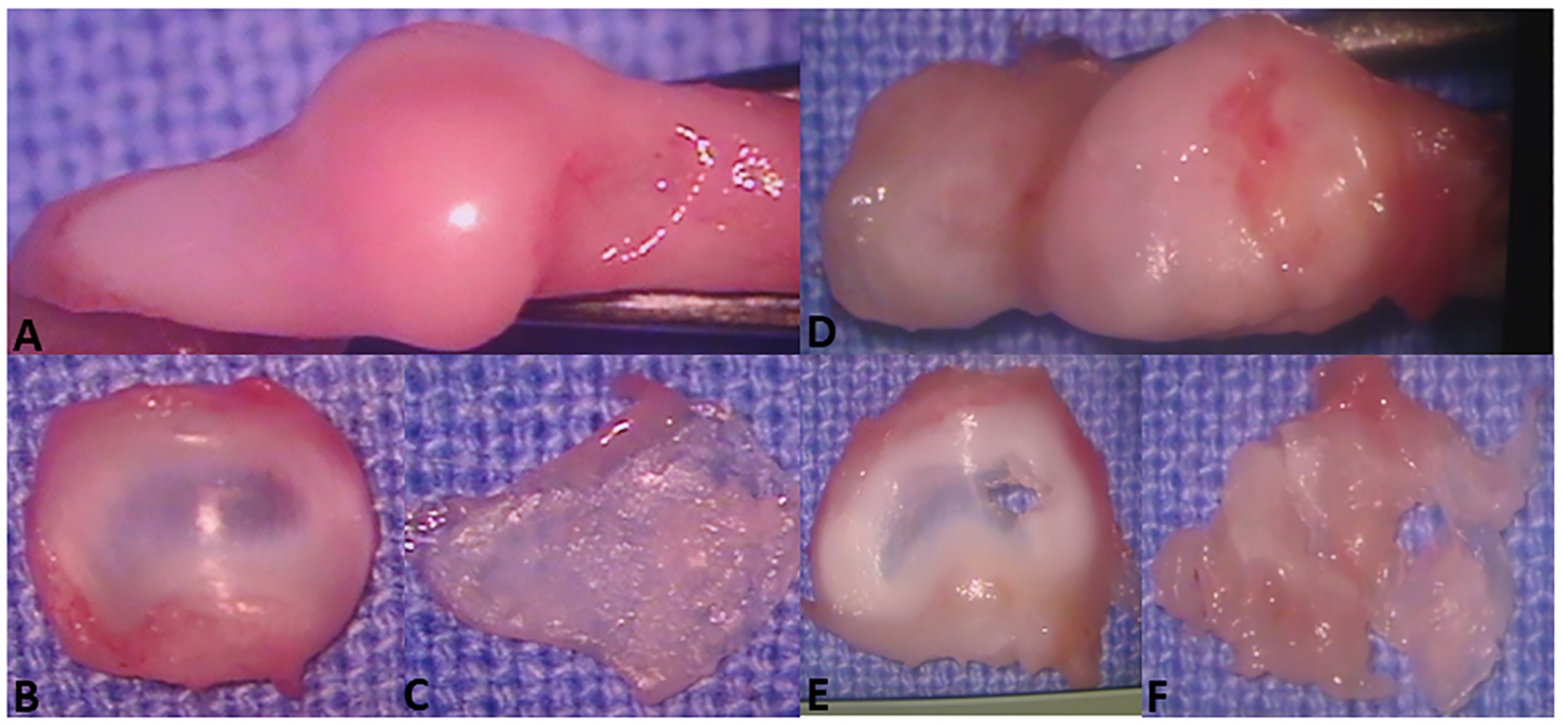
Macroscopic view of temporomandibular joint structures. (A, B, C)—Control group. (A) Condylar surface showing normal convexity and regular contours. (B) Normal disc with characteristic thinning in the central part. (C) Detached condylar cartilage with normal appearance and thickness. Macroscopic view of temporomandibular joint structures. (D, E, F)—Osteoarthritis group. (D) Condylar surface showing erosion, deformation, and flattening. (E) Perforated articular disc. (F) Detached condylar cartilage with perforation and irregularities.

**Fig 4:**
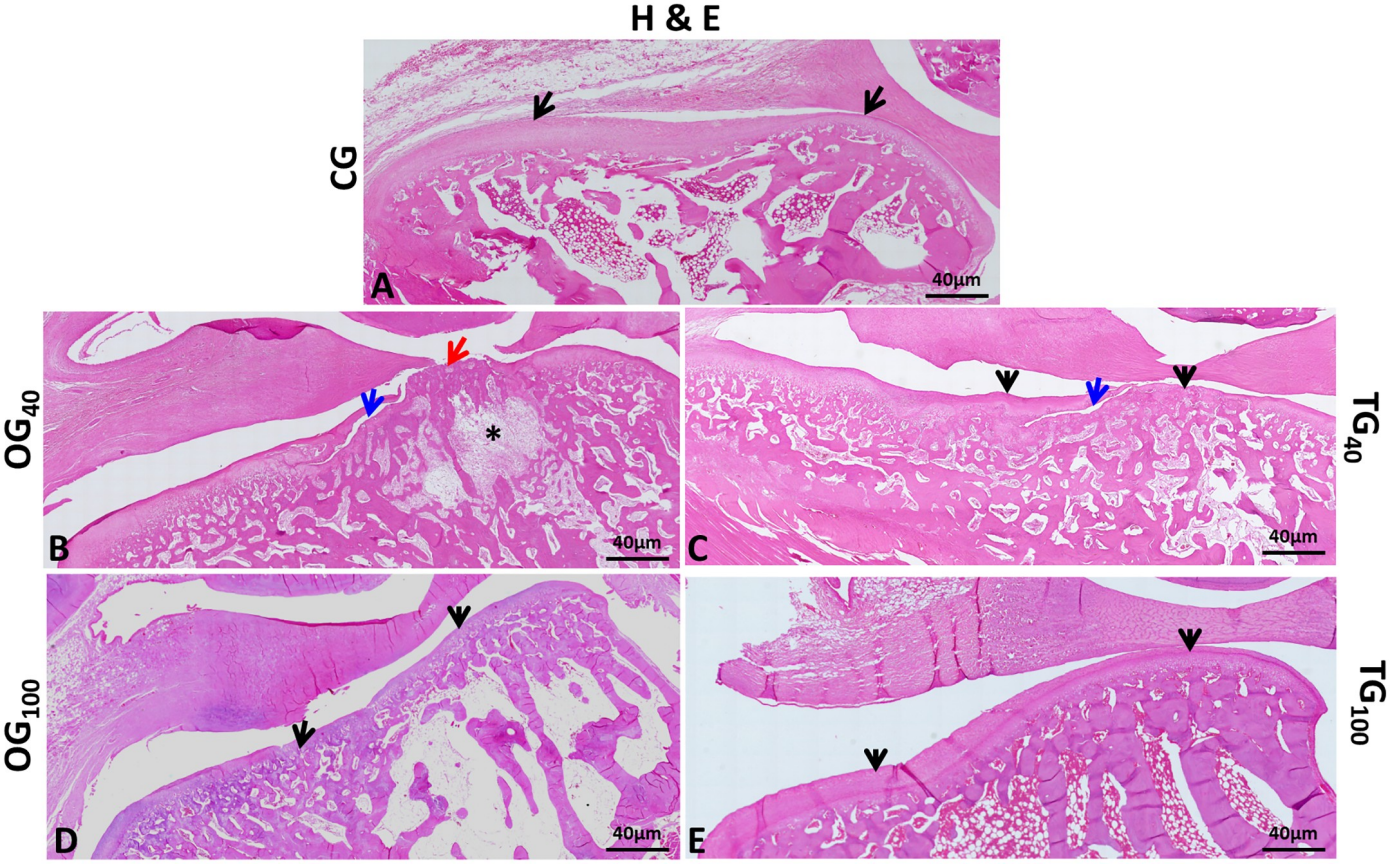
Histologic appearance of the mandibular condyle surface. (A) Control group—TMJ showing normal tissue structures and anatomically normal condyle; characteristic convexity in the anterior region; normal cartilage layer (arrows); intact articular disc. (B and D) OG_40_ and OG_100_—TMJ showing anatomic changes caused by OA. Condylar flattening and deformation; decreased cartilage thickness (black arrows); loss of cartilage matrix (red arrow); crack reaching the subchondral bone tissue (blue arrow); subchondral irregularities; degeneration of bone trabeculae with intra-articular cyst formation (*); tear of the articular disc. (C) TG_40_—Decreased cartilage thickness (black arrows); presence of substantial crack starting at the articular surface and extending in depth (blue arrow); articular disc constricted in the middle. (E) TG100—Tissues with normal appearance; anatomically normal articular condyle; typical condylar convexity in the anterior region; cartilage layer without changes (arrows); intact articular disc. (TMJ: temporomandibular joint, OA: osteoarthritis, OG_40_: osteoarthritis group at 40 days, OG_100_: osteoarthritis group at 100 days, TG_40_: treatment group at 40 days, TG_100_: treatment group at 100 days).

**Fig 5:**
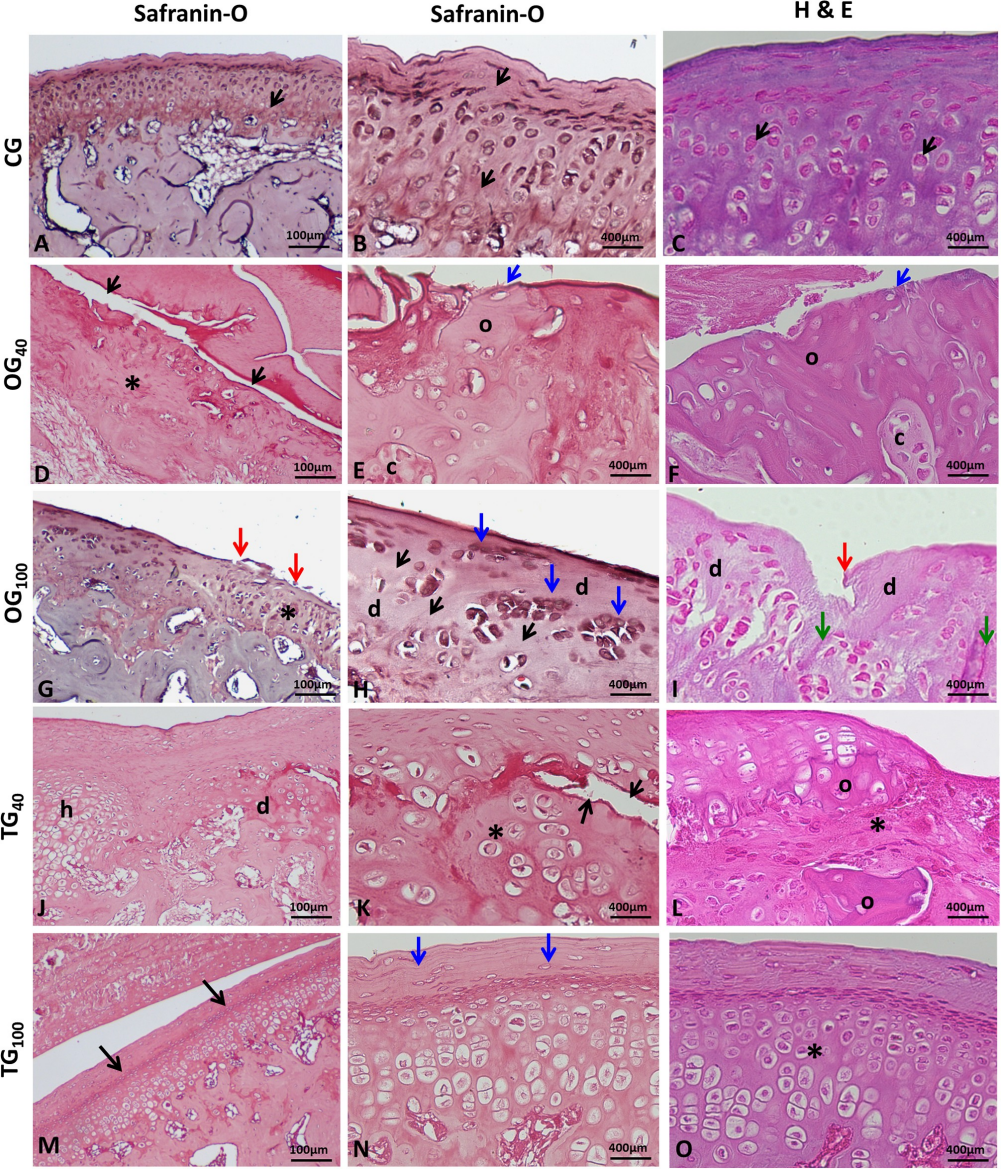
Histologic appearance of the mandibular condyle surface. (A, B, and C) Control group—Normal articular cartilage layer; safranin O staining indicating normal presence of proteoglycans (black arrows); normal presence of chondrocytes and ossification process (red arrows). (D, E, and F) OG40—Ill-defined articular cartilage and cell degeneration (*); presence of cracks (black arrows); loss of cartilage matrix and fibrous connective tissue (blue arrows); areas of superficial heterotopic ossification (o) involving cartilage regions (c). (G, H, and I) OG_100_—Decreased cartilage thickness and subchondral irregularities (*); reduced safranin O staining indicating loss of proteoglycans (black arrows); erosion and discontinuity of cartilage surface (red arrows); cell clustering (blue arrows) close to areas of cell degeneration (d); areas of fibrillation (green arrows). (J, K, and L) TG40—Hypertrophic zone (h) indicating an attempted cartilage repair in the degeneration area (d); deep crack (black arrows); ill-defined cartilage layer (*); heterotopic ossification in degenerative cartilage (o). (M, N, and O) TG_100_—Normal safranin O staining indicating the presence of proteoglycans (black arrows); uniform articular surface and organized fibrous connective tissue (blue arrows); cartilage layer with normal thickness and subdivisions (*). (OG_40_: osteoarthritis group at 40 days, OG_100_: osteoarthritis group at 100 days, TG_40_: treatment group at 40 days, TG_100_: treatment group at 100 days).

CG rabbits showed mandibular condyles with normal morphologic appearance (Fig 3A, 3B and 3C), with a convex anterior region (Fig 4A). Fibrocartilage showed intense safranin O staining in the fibrous connective tissue and underlying hyaline cartilage, with an extracellular cartilaginous matrix without alterations in the molecular composition of proteoglycans (Fig 5A and 5B). Cartilage thickness, with the subdivision of cell layers, and the ossification process were unaltered (Fig 5C).

OG rabbits showed substantial degenerative changes in the TMJ (Fig 3D, 3E and 3F). Morphologically, anatomic modifications were observed in the condyles as a result of subchondral bone tissue remodeling (Fig 4B and 4D). The cartilage layer showed ill-defined structure (Fig 5D), decreased thickness (Fig 5G), and complete cartilage matrix loss in certain areas (Fig 5E), with attempted repair in others. In addition, cartilage surface deformations, deep cracks, and areas of heterotopic ossification were present (Fig 5F). At the cellular level, degeneration (Fig 5H and 5I), attempted cell proliferation, and cell clustering (Fig 5H) were present. The extracellular matrix showed reduced safranin O staining, indicating the loss of proteoglycans. Edema formation was observed in the intercellular region. Intense subchondral bone remodeling was also observed, with the presence of osteoblasts and Howship’s lacunae containing active osteoclasts. There was fibrous connective tissue formation in bone trabeculae. In joints with more severe OA, articular intraosseous cysts indicated advanced degeneration (Fig 4B).

TG_40_ and OG_40_ rabbits showed very similar degenerative changes, with severe OA. However, there was no difference between CG_40_ and TG_40_, indicating joint tissue recovery in the latter. In TG_40_ rabbits, mandibular condyles were deformed, with discontinuity of the cartilage surface by the presence of cracks invading the intermediate and deep layers (Fig 4C). Changes such as fibrillation and erosion were also present. Repair attempts were observed at some sites, as evidenced by areas of cartilaginous tissue formation close to regions of cell degeneration (Fig 5J). Ill-defined cartilage layer (Fig 5K) and heterotopic ossification (Fig 5L) were also observed. In the underlying bone tissue, fibrous connective tissue was observed in the spaces between the bone trabeculae. Most TG_100_ rabbits showed no histologic changes compatible with OA in the TMJ, with morphologically normal condyles and unaltered cartilage thickness (Fig 4E). In some rabbits, joint degeneration was compatible with low grade OA, indicating articular tissue recovery after CS+G treatment. As expected, OG_100_ animals continued to show severe joint degeneration. In TG_100_ rabbits, some condylar regions showed a slight reduction in hyaline cartilage thickness and areas of fibrillation. Eventually, chondrocyte clusters were observed close to areas with reduced cell numbers. There was normal safranin O staining in the articular cartilage, suggesting the recovery of proteoglycans in the extracellular matrix (Fig 5M). Overall, the cartilage layer and subchondral bone had a histologic appearance without evidence of degenerative changes (Fig 5N and 5O).

### GAG quantification

Considering the time points, no significant changes were observed in GAG content in the articular discs (F = 0.645; *p = 0*.*667*) and cartilage (F = 1.892; *p = 0*.*125*) between the 3 groups. Without considering the time points, there was a significant increase in GAG concentrations in the articular discs after OA induction (OG: 4.04±1.37 vs CG: 3.02±0.82, *p = 0*.*041*) (Table 1). However, there was no significant difference between CG (3.02±0.82) and TG (3.64±1.54), suggesting that GAGs returned to near normal levels in the articular discs of treated rabbits (*p = 0*.*241*) (Table 1). The same analysis in the articular cartilage showed no significant difference between CG (7.57±4.25) and OG (8.78±3.73) (*p = 0*.*468*) or between CG (7.57±4.25) and TG (8.83±2.61) (*p = 0*.*391*). (S2 Table).

**Table 1:** Concentration of glycosaminoglycans in control vs osteoarthritis groups and control vs treatment groups.

*Group*	*Structure*			
	*Disc*	*Cartilage*	*Disc*	*Cartilage*
*Control*	*3*.*02±0*.*82*	*7*.*57±4*.*25*	*3*.*02±0*.*82*	*7*.*57±4*.*25*
*Osteoarthritis*	*4*.*04±1*.*37*	*8*.*78±3*.*73*		
*Treatment*			*3*.*64±1*.*54*	*8*.*83±2*.*61*
*p (< 0*.*05)*	*0*.*041*	*0*.*468*	*0*.*241*	*0*.*391*

*mean ± standard deviation (μg/mg wet weight)

## Discussion

Treated rabbits had no adverse effects and tolerated well the medication, handling, and route of administration used. In addition, the diet offered was well accepted, leading to significant weight gain at all assessment time points. Our data show a late beneficial effect of CS+G administered subcutaneously on TMJ tissues in rabbits with OA. Oral CS+G is widely used as a supplement by patients with OA and has been tested in several experimental animal studies [11,13,40]. However, oral administration to rabbits would require the preparation of rabbit-specific foods containing the active ingredient or gavage administration. Disadvantages include the high cost of diet preparation and potential iatrogenic complications resulting from long-term gavage administration. In addition, animal handling to perform the technique could become difficult over time, precluding administration. Therefore, the subcutaneous route was chosen for practicality and safety purposes, providing a successful route of administration until the end of the experiment.

The rabbit TMJ is anatomically and physiologically similar to the human TMJ [41]. The incomplete glenoid fossa in the posterior region also facilitates access for experimental manipulation. An example of this advantage is the possibility to inject substances intra-articularly without the need to access the joint surgically, and this is one of the reasons why rabbit models have emerged as the animal models of choice for the experimental study of diseases and therapies in the human TMJ [42,43]. In addition, we decided to inject MIA directly into the rabbit joint cavity given the results of previous studies confirming the efficacy of OA induction in the TMJ and important late degenerative changes [36,41,44].

Late beneficial effects of CS+G were observed on TMJ tissues with OA. Previous studies of CS+G treatments have shown positive results in improving symptoms [32,35]. However, the authors are unaware of a previous study that showed positive effects of CS+G used as DMOADs in TMJ-OA in an animal model. CS+G had an anabolic effect on the articular cartilage by promoting extracellular matrix production, suppressing inflammatory mediators, and inhibiting tissue degeneration [45]. It should be noted that there was a large variability in the histologic data of animals subjected to OA, especially in the first 40 days after disease induction. These data reflect a period of marked adaptation of the tissues involved in OA [38]. Moreover, after 30 days of treatment, there was no improvement in OA in TG_40_ compared with OG_40_. After injection of MIA, peak matrix metalloproteinase (MMP) levels and aggrecanase proteoglycan cleavage sites occur between days 3 and 7 [46]. In the rat knee, inflammatory processes and histologic effects have been observed by day 1 and 7 post-MIA injection, respectively [47]. In the TMJ, decreased cartilage thickness and presence of subchondral bone invasion have been observed by day 10 post-MIA injection [41]. This was the waiting time to start treatment with CS+G after MIA injection in the present study. The action of CS+G as a symptomatic slow-acting drug for OA (SYSADOA) begins at 2 to 3 weeks after the start of use [48]. A study evaluating oral CS and G used as DMOADs in the rabbit femur for defect filling with implantation of autologous cultured chondrocytes reported cell cluster formation and columnar arrangements at 12 weeks. At 24 weeks, cell columns and substantial extracellular matrix containing proteoglycans and type II collagen were observed. The effect of CS and G without autologous chondrocyte implantation on the treatment of cartilage defects was unconvincing [13]. However, in the present study, rabbits showed an improvement in OA severity after 30 days of CS+G treatment (OG_40_ vs TG_40_).

Although the pathophysiologic mechanisms by which CS+G acts on OA have not been fully elucidated [49], the treatment tested here yielded positive results in TMJ tissues damaged by OA after 90 days of treatment (TG_100_). Treated rabbits showed a significantly reduced degeneration (OG_100_ vs TG_100_), an effect that we attribute to the action of CS+G as a DMOAD. However, a similar treatment effect was observed over time (TG_40_ vs OG_100_). Taşkesen et al. [40] observed a repair process in the extensor digitorum longus tendon of rabbits subjected to surgical lesion after treatment with CS+G. There was increased cartilage formation and decreased formation of blood vessels after 6 weeks of treatment. However, these differences were not observed in comparison with the control group after 12 weeks of CS+G use. The treated group also showed tissue improvement, similar to that observed in the present study after 90 days of treatment. Roman-Blas et al. [11] induced OA in the rabbit knee by cruciate ligament transection and partial medial meniscectomy and tested the oral administration of CS+G for 14 weeks. Histologic parameters showed no reduction in disease severity in the hyaline cartilage that could indicate a therapeutic effect of CS+G as a DMOAD. In addition, long-term use of CS+G did not reduce the high levels of the inflammatory cytokines IL-1β and COX2 present in the synovial membrane of animals with OA. However, in a rabbit model of arthritis, intraperitoneal G administration had an effect on the modulation of the inflammatory process in the knee synovial membrane, with less intense mononuclear cell infiltration than that of control animals [14]. It should be taken into consideration that the hyaline cartilage structure lacks blood supply, does not contain fibrous connective tissue and has a limited number of progenitor cells. Nevertheless, 60-day combined CS+G treatment in female rats subjected to tibial epiphyseal degeneration by ovariectomy resulted in marked chondrocyte proliferation and significant longitudinal bone growth [50]. In the TMJ, however, fibrocartilage may behave similarly to the perichondrium by becoming a source of mesenchymal cells that are precursors of new chondrocytes [51]. Therefore, it can be suggested that the effect of CS+G associated with the characteristics of fibrocartilage may have promoted the regression of the TMJ degenerative process observed in the present study.

Previous studies have reported complete loss of extracellular matrix GAGs both in articular cartilage with severe OA [52,53] and in degenerative or displaced articular discs [54]. However, in the present study, there was a significant increase in GAGs in the articular discs of OG vs CG rabbits, when analyzing the 2 time points together. This result was also reported by Axelsson [55] in a study in which OA was induced by surgical disc perforation in the TMJ of rabbits. After 16 weeks, larger amounts of GAGs were detected in treated rabbits than in controls. In experimental articular discs, the population of large proteoglycans had a slightly increased synthesis and decreased degradation rate. In the early stage of OA or initial repair stage, the increased synthesis of the extracellular matrix and DNA components characterized by cell proliferation and clustering occurs biochemically, resulting in an increased metabolic activity of chondrocytes [56], which may explain the results of the present study. In an *in vitro* study, chondrocytes responded with increased GAG release in the presence of interleukin 1 (IL-1) [57]. A positive effect has also been observed on GAG production by chondrocytes cultured in fibrin-alginate hydrogel supplemented with CS and hyaluronic acid. Supplemented hydrogels have also shown significantly higher cellular DNA levels than non-supplemented control hydrogels [58]. Thus, in the OA model used here, it could be suggested that chondrocytes responded with increased GAG production. However, such processes occur in the cartilage, where the results differed from those observed in the disc. There was no difference in GAG concentration between CG and OG, which suggests that OA may induce greater changes in the cartilage, reducing its responsiveness to disease—even though repair attempts were observed in some cartilage regions, with cell proliferation and clustering, which might have led to an increased production of molecular components, such as GAGs. The attempted repair process is mediated by growth factors, which diffuse through the extracellular matrix to reach the chondrocytes. In OA, there is a greater-than-normal diffusion of growth factors resulting from loss of tissue integrity [55].

No significant difference was found in mean articular disc GAG concentration between CG and TG rabbits, considering the 2 assessment time points. This result suggests that the improvement in OA severity, as a result of CS+G treatment, may have led to a decrease in GAG release by chondrocytes. CS and G exert an effect by enhancing the response of chondrocytes to adverse environmental conditions, such as in the presence of OA, but not in normal cartilage [59]. These agents stimulate extracellular matrix turnover by increasing GAG production in the presence of OA, with subsequent normalization of the metabolism, thus returning molecular components to normal levels [52]. *In vitro* studies have shown that the presence of IL-1 induces the release of GAGs and that both G sulfate alone and the combination of high doses of CS+G can reduce the release of these molecular components by chondrocytes in joint tissues [57,59]. However, in the present study, there was no significant difference in articular cartilage GAG concentration between CG and TG rabbits. A possible explanation is that the differences in GAG contents between articular discs and cartilages may result from the lack of specificity of DMB reaction due to GAG metabolism byproducts or the lack of sensitivity of the assay [57].

The model provided in the present study is easily reproducible, as it obviates the need for surgical procedures. In addition, the results obtained with the CS+G treatment support the conclusion that CS+G can reverse degenerative changes in the intra-articular structures of the TMJ, which makes it possible to regard them as DMOADs. However, in the treatment of TMJ-OA, other factors that can interfere with the pathogenesis of the disease should also be considered, especially age, sex, dental occlusion, and behavioral habits. Moreover, because of the limited repair capacity of cartilage and the complex mechanisms that lead to the development of OA, additional studies are needed to further evaluate the effect of CS+G on joint tissues with OA and their mechanisms of action as DMOADs.

## Supporting information

S1 Table Osteoarthritis degree. (XLSX)

S2 Table GAG’s quantification. (XLSX)

S1 Checklist (DOCX)
