# Supplementary material for: Reduction of osteoarthritis severity in the temporomandibular joint of rabbits treated with chondroitin sulfate and glucosamine
Source: PLoS One. 2020 Apr 15;15(4):e0231734. doi: 10.1371/journal.pone.0231734 (PMC7159193; doi:10.1371/journal.pone.0231734)

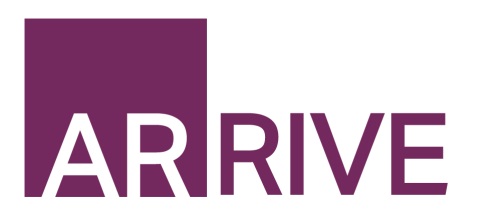


The ARRIVE Guidelines Checklist

Animal Research: Reporting In Vivo Experiments

Carol Kilkenny^1^, William J Browne^2^, Innes C Cuthill^3^, Michael Emerson^4^ and Douglas G Altman^5^

*^1^The National Centre for the Replacement, Refinement and Reduction of Animals in Research, London, UK, ^2^School of Veterinary Science, University of Bristol, Bristol, UK, ^3^School of Biological Sciences, University of Bristol, Bristol, UK, ^4^National Heart and Lung Institute, Imperial College London, UK, ^5^Centre for Statistics in Medicine, University of Oxford, Oxford, UK.*

|  | | ITEM | RECOMMENDATION | Section/ Paragraph |
| --- | --- | --- | --- | --- |
| 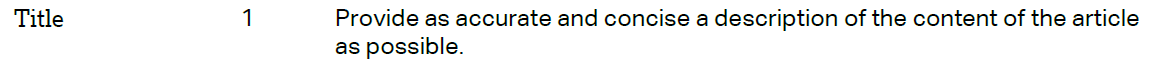 | | | Title page |  |
| 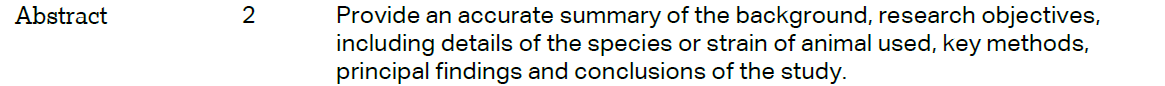 | | | Page 3 |  |
| INTRODUCTION | | |  |  |
| 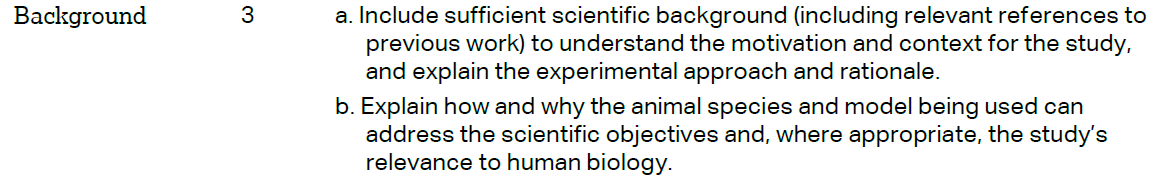 | | | Introduction  paragraphs 2 and 3 |  |
| 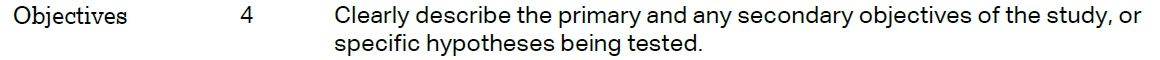 | | | Introduction  paragraph 4 |  |
| METHODS | | |  |  |
| 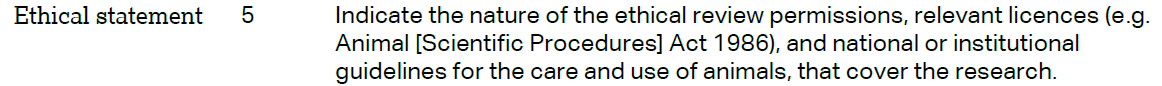 | | | Methods  paragraph 1 |  |
| 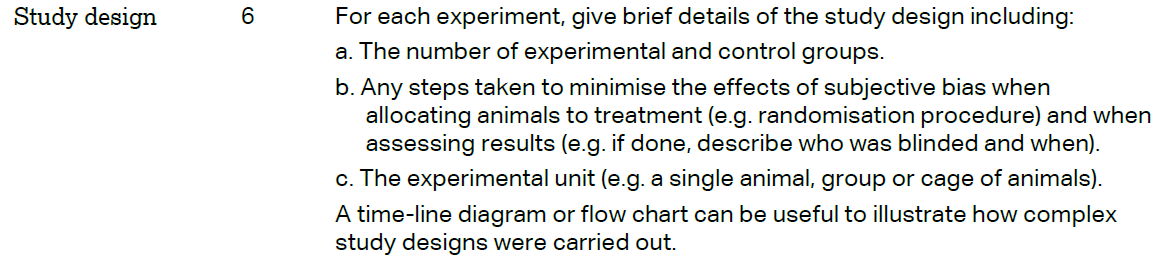 | | | Methods  Experimental design:  Paragraphs 1 and 2 |  |
| 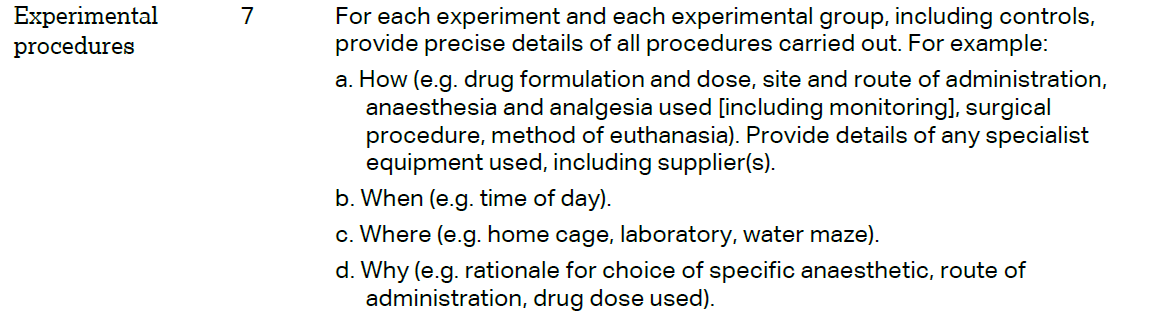 | | | Materials and  Methods  Treatment, histological analysis and preparation of samples and quantification of GAGs  paragraphs 3, 4 and 5 |  |
| 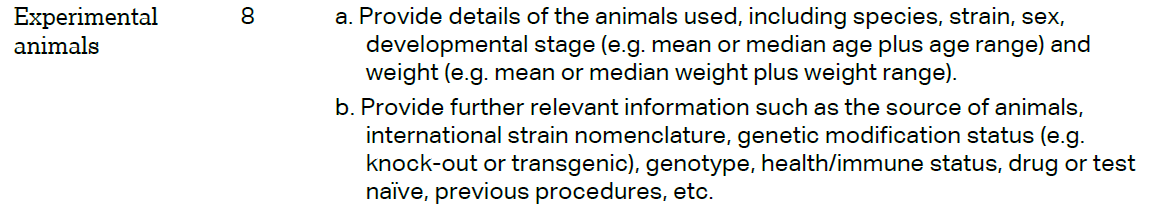 | | | Materials and  Methods  Experimental design:  paragraph 1 |  |

The ARRIVE guidelines. Originally published in *PLoS Biology*, June 2010^1^

| 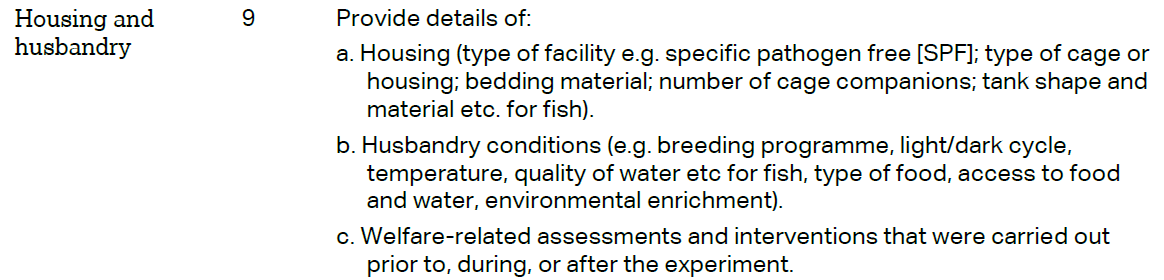 | Materials and  Methods  Experimental design:  paragraph 1 | |
| --- | --- | --- |
| 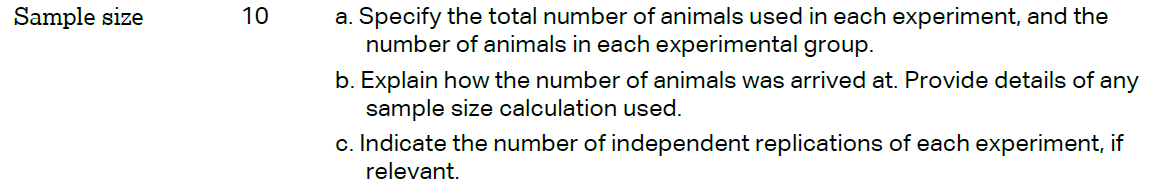 | Materials and  Methods  Experimental design:  paragraphs 1 and 2 | |
| 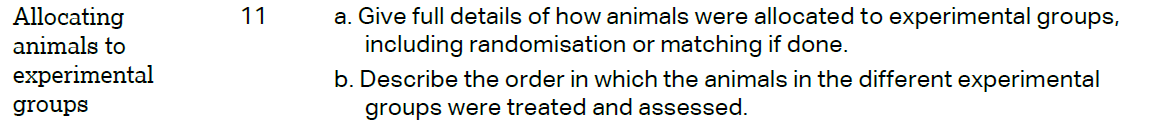 | Materials and Methods  Experimentaldesign:  paragraph 2 | |
| 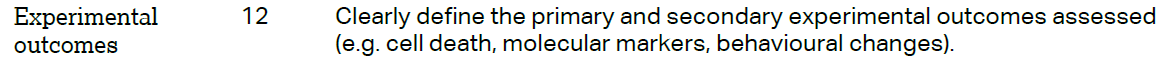 | Materials and Methods  Treatment, histological analysis and preparation of samples and quantification of GAGs  paragraph 3, 4 and 5 | |
| 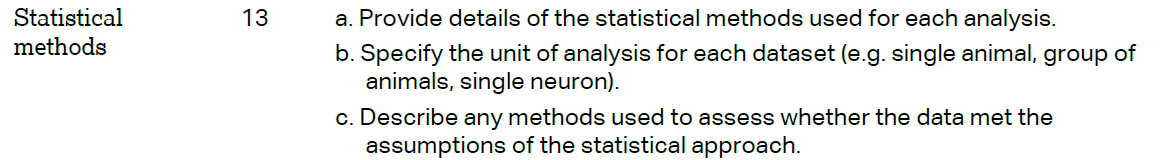 | Materials and Methods  Statistical analysis  paragraph 6 | |
| RESULTS |  | |
| 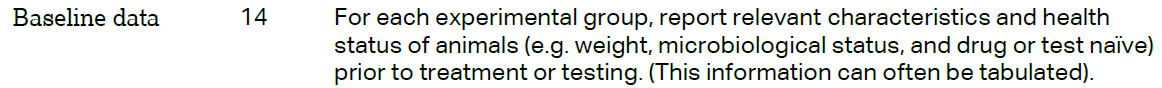 | Results  Severity of Ostearthritis:  paragraph 1 | |
| 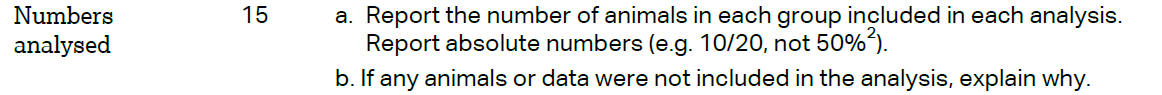 | Materials and Methods  Experimental design:  paragraph 1 | |
| 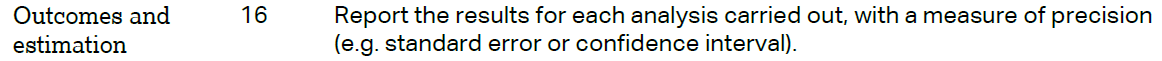 | Results  Severity of OA, histological evaluation and quantification of GAGs  paragraphs 1, 2 and 3 | |
| 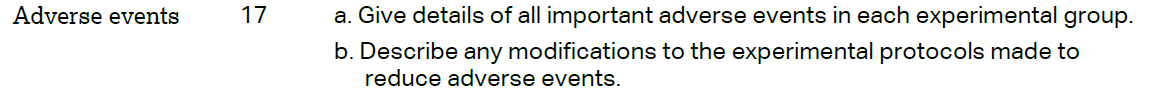 | No adverse events occurred | |
| DISCUSSION |  | |
| 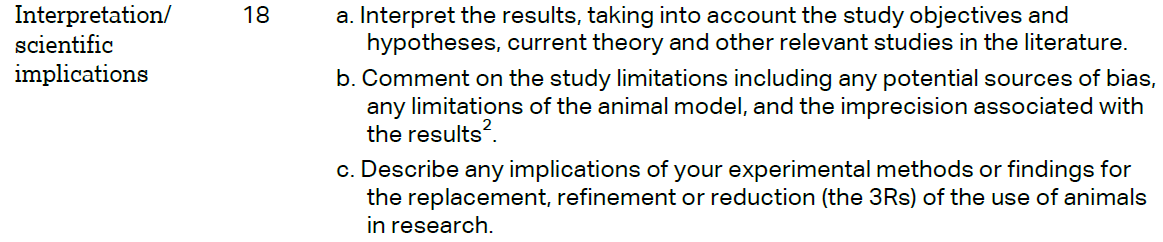 | Discussion  paragraphs 1-6 | |
| 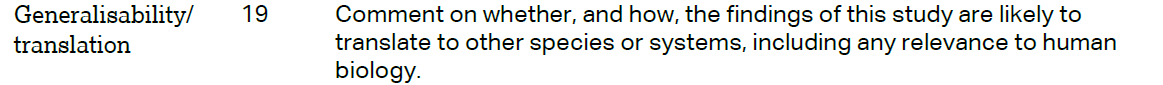 | Discussion  paragraph 6 | |
| 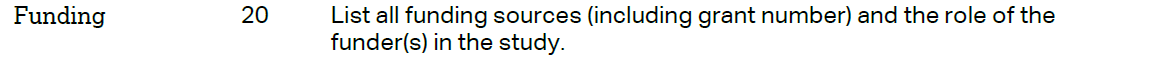 | | Acknowledgements  page 17 |


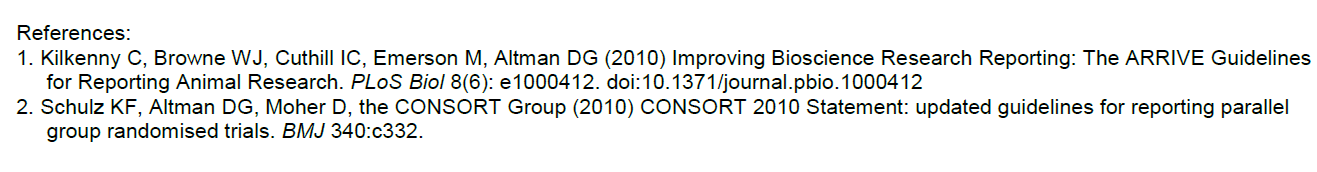

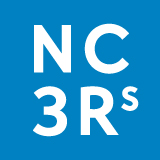

Supplement: S1 Checklist — (DOCX) [file pone.0231734.s003.docx]
